# Early Versus Postoperative Chemical Thromboprophylaxis Is Associated with Increased Bleeding Risk Following Abdominal Visceral Resections: a Multicenter Cohort Study

**DOI:** 10.1007/s11605-022-05301-4

**Published:** 2022-03-22

**Authors:** David S. Liu, Ryan Newbold, Sean Stevens, Enoch Wong, Jonathan Fong, Krinal Mori, Darren J. Wong, Anna Sonia Gill, Sharon Lee, Wael Jamel, Amy Crowe, Tess Howard, Anshini Jain, Pith Soh Beh, Maeve Slevin, Nicola Fleming, Simon Bennet, Chi Chung, VERITAS collaborative

**Affiliations:** 1 Division of Surgery, Anaesthetics and Procedural Medicine, Austin Health, 145 Studley Road, Heidelberg, Victoria 3084 Australia; 2 Department of Surgery, Northern Health, 185 Cooper Street, Epping, Victoria 3076 Australia; 3 Department of Surgery, The University of Melbourne, Austin Health, 145 Studley Road, Heidelberg, Victoria 3084 Australia; 4 Division of Cancer Surgery, Peter MacCallum Cancer Centre, 305 Grattan Street, Melbourne, Victoria 3000 Australia; 5 General and Gastrointestinal Surgery Research Group, Austin Precinct, Department of Surgery, The University of Melbourne, 145 Studley Road, Heidelberg, Victoria 3084 Australia; 6 Department of Surgery, Box Hill Hospital, 8 Arnold Street, Box Hill, Victoria 3128 Australia; 7 Department of Surgery, The University of Melbourne, Northern Health, 185 Cooper Street, Epping, Victoria 3076 Australia; 8 Department of Gastroenterology, Austin Health, 145 Studley Road, Heidelberg, Victoria 3084 Australia

**Keywords:** Thromboembolism, Chemoprophylaxis, Timing, Laparotomy

## Abstract

**Background:**

Abdominal visceral resections incur relatively higher rates of postoperative bleeding and venous thromboembolism (VTE). While guidelines recommend the use of perioperative chemical thromboprophylaxis, the most appropriate time for its initiation is unknown. Here, we investigated whether early (before skin closure) versus postoperative commencement of chemoprophylaxis affected VTE and bleeding rates following abdominal visceral resection.

**Methods:**

Retrospective review of all elective abdominal visceral resections undertaken between January 1, 2018, and June 30, 2019, across four tertiary-referral hospitals. Major bleeding was defined as the need for blood transfusion, reintervention, or > 20 g/L fall in hemoglobin from baseline. Clinical VTE was defined as imaging-proven symptomatic disease < 30 days post-surgery.

**Results:**

A total of 945 cases were analyzed. Chemoprophylaxis was given early in 265 (28.0%) patients and postoperatively in 680 (72.0%) patients. Mean chemoprophylaxis exposure doses were similar between the two groups. Clinical VTE developed in 14 (1.5%) patients and was unrelated to chemoprophylaxis timing. Postoperative bleeding occurred in 71 (7.5%) patients, with 57 (80.3%) major bleeds, requiring blood transfusion in 48 (67.6%) cases and reintervention in 31 (43.7%) cases. Bleeding extended length-of-stay (median (IQR), 12 (7–27) versus 7 (5–11) days, *p* < 0.001). Importantly, compared to postoperative chemoprophylaxis, early administration significantly increased the risk of bleeding (10.6% versus 6.3%, RR 1.45, 95% CI 1.05–1.93, *p* = 0.038) and independently predicted its occurrence.

**Conclusions:**

The risk of bleeding following elective abdominal visceral resections is substantial and is higher than the risk of clinical VTE. Compared with early chemoprophylaxis, postoperative initiation reduces bleeding risk without an increased risk of clinical VTE.

## Introduction

Patients undergoing major abdominal visceral resection are at risk of both venous thromboembolism (VTE) and postoperative bleeding.^1^ Both perioperative complications cause significant morbidity and mortality and contribute to the burden of healthcare costs. International guidelines recommend the use of both pharmacological and mechanical strategies to prevent perioperative VTE.^2,3^ While these approaches have proven efficacious in the prevention of postoperative VTE, chemical thromboprophylaxis, in particular, may confer an increased risk of postoperative bleeding.^2^ Fundamentally, the use of chemical thromboprophylaxis must be balanced against the risk of bleeding in order to reduce complications and improve patient outcomes.

The use of chemoprophylaxis is common place in surgical practice. Multiple guidelines exist that support their use.^2,3^ Despite this, there is a paucity of data regarding optimal timing of chemoprophylaxis in the perioperative period. Significant variations in practice exist in regard to the time at which chemoprophylaxis is commenced. This has been shown in multicenter studies by the PROTECTinG (Perioperative Timing of Elective Chemical Thromboprophylaxis in General surgery) investigators within the VERITAS (Victorian-collaborative for Education, Research, Innovation, Training and Audit by Surgical trainees) collaborative.^1,4,5^ Within this general surgical cohort, there is particular heterogeneity amongst patients undergoing major abdominal visceral resections.^1^ Timing of chemoprophylaxis has been shown to affect the risk of bleeding in other general surgical procedures such as cholecystectomy and breast surgery.^5,6^ We believe that poorly timed chemoprophylaxis may result in increased patient morbidity, due to either postoperative bleeding or VTE. Therefore, it is critical that we investigate the variation in perioperative chemoprophylaxis and establish an evidence base that guides optimal timing, particularly in patients undergoing major abdominal visceral resection.

Several studies have published data on the timing of chemoprophylaxis in patients undergoing major abdominal surgery. These have largely focused on liver, pancreas, biliary, and colorectal operations^7–11^. However, these studies are typically single surgeon or single center in origin^8–10^ and are underpowered to detect a true difference in the incidence of bleeding and/or VTE.^8–10^ They include a heterogenous group of procedures with vastly different bleeding and VTE risk profiles^9^ and often use asymptomatic VTE as an endpoint.^7^ One also includes the testing of an experimental agent that has not translated into use routine clinical practice.^11^ Given these limitations, there has not been a consensus to guide optimal timing of perioperative chemoprophylaxis in patients undergoing major abdominal visceral resection.

In this multicenter cohort study, we aim to investigate the timing of chemoprophylaxis, given early before skin closure versus postoperatively, in patients undergoing major abdominal visceral resections, and the effect this has on rates of bleeding and symptomatic VTE.

## Methods

### Study Design

We retrospectively analyzed consecutive patients who underwent elective major abdominal visceral resections from January 1, 2018, to June 30, 2019, at four Victorian tertiary-referral centers in Australia. These include the Northern, Austin, Box Hill, and Maroondah hospitals. Overall, surgeries were performed by 41 consultant surgeons and 20 trainees. Patients were identified from each hospital’s administrative database using the Australian Classification of Health Interventions procedural code for esophagectomy, gastrectomy, splenectomy, hepatectomy, pancreatectomy, duodenectomy, small bowel resection, colectomy, and proctectomy.^1^ These procedures were chosen as they shared a similar bleeding and VTE risk profile,^1^ and patients were expected to stay in hospital for greater than two postoperative days. We excluded patients under 18 years of age, stand-alone cholecystectomy, and bariatric and emergency procedures. This study was approved by the Human Research Ethics Committee across all centers.

### Venous Thromboembolism Prophylaxis

Mechanical thromboprophylaxis included both sequential compression devices and graduated compression stockings. Chemoprophylaxis involved subcutaneous injection of enoxaparin (daily), heparin (twice daily), or dalteparin (daily), at doses adjusted to each patient’s creatinine clearance and weight. We classified the timing of chemoprophylaxis into two groups: given before (early) or after (postoperative) skin closure. The type and timing of chemoprophylaxis was at the discretion of the treating team.

### Data Collection and Quality Assurance

Data from medical records was extracted onto a universal electronic proforma. This included patient demographics, comorbidities, perioperative parameters, operative details, timing of chemoprophylaxis, postoperative bleeding, and VTE events. Quality assurance measures to maximize data accuracy and minimize inter-observer discrepancies included the use of a standardized data collection tool, binary or quantitative data fields, training sessions for data collectors, and exclusion of patients with > 5% incomplete data. A random audit of 10% of data fields demonstrated a mean accuracy rate of 98.0% (range: 96.3–99.1%).

### Study Endpoints and Definitions

Our primary endpoint is the incidence of postoperative bleeding. Secondary endpoints include length-of-stay, changes in hemoglobin post-surgery, major and minor bleeding events, need for blood transfusion and reintervention to achieve hemostasis, 30-day clinical VTE, overall complication, and postoperative mortality rates. We defined postoperative bleeding as any bleeding that occurred within the same admission period. Major bleeding was defined as the need for blood transfusion, reintervention (surgical or radiological), or a > 20-g/L fall in hemoglobin from baseline.^5^ Minor bleeding was defined as any bleeding event that did not meet major bleeding criteria. Clinical VTE was defined as radiologically proven (computer tomography pulmonary angiography, ventilation-perfusion scintigraphy, and/or venous duplex ultrasound) symptomatic disease within 30-day post-resection. Surgical complications were graded according to the Clavien-Dindo classification.^12^ The VTE risk for each patient was calculated using the Caprini score (≤ 2: low, 3–4: moderate, ≥ 5: high risk).^13^ As per local hospital guidelines, oral antiplatelet and anticoagulant agents, excluding aspirin, were withheld 3–7 days pre-surgery. Patients who required ongoing therapeutic anticoagulation were bridged with enoxaparin up to 24 h before surgery. All patients were followed up in clinic between 4 and 6 weeks post-discharge.

### Power Calculation

From our initial studies,^1^ an 11% risk of bleeding following major abdominal visceral resection was used for power calculation. We deemed a ≥ 4% absolute difference between study groups as clinically significant. Given the approximate ratio of 1:2 for early versus postoperative chemoprophylaxis usage in our cohort,^1^ the total sample size required was 900 cases (early: 300, postoperative: 600) to achieve 80% statistical power (alpha < 0.05).

### Statistical Analysis

Continuous and categorical variables were analyzed using the Student’s *t*-test and Fisher’s exact test, respectively. To determine independent predictors of postoperative bleeding, and account for differences in hospital-based practices, a hierarchical multivariate logistic regression analysis was undertaken. In this model, covariates were treated as fixed effects, whereas hospital was treated as a random effect. A two-tailed *p* < 0.05 and 95% confidence interval (CI) around the odds ratio (OR) that did not cross one was considered statistically significant. Statistical analyses were conducted using Prism v9 (GraphPad Software, San Diego, CA, USA) and R v4.1.0 (R Foundation for Statistical Computing, Vienna, Austria).

## Results

### Baseline Characteristics Between Early and Postoperative Chemoprophylaxis Groups

In total, abdominal visceral resection was undertaken in 945 patients. Chemoprophylaxis was commenced early in 265 (28.0%) patients and postoperatively in 680 (72.0%) patients. The number of chemoprophylaxis exposure doses was similar between the two groups (median (IQR), early: 7 (5–13), postoperative: 7 (4–10), *p* = 0.13). For patients who only received postoperative chemoprophylaxis, the median time from skin closure to anticoagulant injection was 8.4 (IQR 6.1–15.7) h.

Both early and postoperative chemoprophylaxis groups were comparable with regard to demographic, operative, and perioperative characteristics (Table 1). In particular, they shared similar surgical case mix, operator experience, use of mechanical thromboprophylaxis, preoperative antiplatelet and anticoagulant consumption, complication grade, and length-of-stay. The early chemoprophylaxis group had a higher proportion of open surgery which, overall, took longer to perform.

**Table 1:** Baseline characteristics between early and post-op chemoprophylaxis groups

Characteristics	Early *N* = 265	Post-op *N* = 680	Early vs. Post-op *p*-value
Demography			
Gender, male, *n* (%)	136 (51.3)	340 (50.0)	0.72
Age, mean (SD)	62.0 (14.1)	64.0 (12.3)	0.15
Body mass index (kg/m^2^), mean (SD)	28.3 (6.3)	28.2 (6.2)	0.93
Caprini score, median (IQR)	6 (5–7)	6 (5–7)	0.53
Operative			
Type, *n* (%)			0.56
Esophagectomy	10 (3.8)	24 (3.5)	
Gastrectomy	15 (5.7)	15 (2.2)	
Splenectomy	3 (1.1)	10 (1.5)	
Liver resection	56 (21.1)	77 (11.3)	
Bile duct resection	4 (1.5)	8 (1.2)	
Pancreatectomy	49 (18.5)	60 (8.8)	
Small bowel resection	10 (3.8)	21 (3.1)	
Colon resection	47 (17.7)	197 (29.0)	
Rectal resection	61 (23.0)	249 (36.6)	
Hartmann reversals	10 (3.8)	19 (2.8)	
Surgeon level, *n* (%)			0.40
Consultant	255 (96.2)	644 (94.7)	
Trainee	10 (3.8)	36 (5.3)	
Approach, *n* (%)			**0.017**
Open	116 (43.8)	239 (35.1)	
Laparoscopic	149 (56.2)	441 (64.9)	
Surgery length (min), mean (SD)	358.4 (172.0)	257.8 (123.3)	** < 0.001**
Perioperative			
Malignant pathology, yes, *n* (%)	196 (74.0)	466 (68.5)	0.11
Therapeutic anticoagulant use, yes, *n* (%)	19 (7.2)	44 (6.5)	0.67
Antiplatelet agents use, yes, *n* (%)	29 (10.9)	103 (15.1)	0.10
ASA score, median (IQR)	3 (2–3)	3 (2–3)	0.34
Mechanical prophylaxis use, yes, *n* (%)	263 (99.2)	678 (99.7)	0.31
Chemoprophylaxis exposure dose, median (IQR)	7 (5–13)	7 (4–10)	0.13
Pre-op hemoglobin (g/L), mean (SD)	138.5 (87.4)	132.2 (19.5)	0.25
Pre-op platelet count, × 10^9^/L, mean (SD)	274.4 (101.3)	274.5 (99.9)	0.99
Pre-op INR, unit, mean (SD)	1.1 (0.1)	1.1 (0.2)	0.10
Pre-op bilirubin, mmol/L, mean (SD)	11.3 (18.9)	9.3 (8.2)	0.11
Pre-op albumin, g/L, mean (SD)	37.2 (4.8)	37.6 (6.0)	0.29
Clavien-Dindo ≥ 3, *n* (%)	28 (10.6)	70 (10.3)	0.91
Hospital stays, days, median (IQR)	8 (6–14)	7 (5–11)	0.11

*ASA* American Society of Anesthesiology, *INR* international normalize ratio, *IQR* interquartile range, *SD* standard deviation

Bold indicates statistical significance

### Clinical Venous Thromboembolism Following Abdominal Visceral Resections

Fourteen (1.5%) patients developed clinical VTE within 30 days post-surgery. Of these, 4 had deep vein thromboses, 6 had pulmonary embolisms, and 4 had concurrent presentations. These events occurred in patients at moderate and high risk of VTE. The rate of clinical VTE was not significantly different between those who received early (2.6%) and postoperative (1.0%) chemoprophylaxis (*p* = 0.10; Table 2).

**Table 2:** Bleeding and VTE outcomes in overall cohort

Outcomes	Early *N* = 265	Post-op *N* = 680	Early vs. Post-op		
			OR	95% CI	*p*-value
All bleeding, *n* (%)	28 (10.6)	43 (6.3)	1.75	1.08–2.89	**0.038**
Major bleeding, *n* (%)	25 (9.4)	32 (4.7)	2.11	1.25–3.40	**0.009**
Minor bleeding, *n* (%)	3 (1.1)	11 (1.6)	0.70	0.21–2.25	0.77
Surgery for bleeding, *n* (%)	16 (6.0)	15 (2.2)	2.85	1.43–5.79	**0.007**
Blood transfusion for bleeders, *n* (%)	19 (7.2)	29 (4.3)	1.73	0.94–3.11	0.07
Blood transfusion overall, *n* (%)	42 (15.8)	56 (8.2)	2.10	1.38–3.21	**0.001**
Hb drop for bleeders, g/L, mean (SD)	− 27.8 (19.8)	− 25.2 (19.8)	-	-	0.59
Hb drop overall, g/L, mean (SD)	− 22.5 (15.4)	− 16.6 (14.9)	-	-	** < 0.001**
Venous thromboembolism, *n* (%)	7 (2.6)	7 (1.0)	2.61	0.98–6.96	0.08

*CI* confidence interval, *Hb* hemoglobin, *OR* odds ratio, *SD* standard deviation

Bold indicates statistical significance

### Early Chemoprophylaxis Increases Bleeding Risk Following Abdominal Visceral Resections

Bleeding was identified in 71 (7.5%) patients (Table 2). Of these, 57 (80.3%) were major events. Intra-abdominal hemorrhage was the most common etiology (64 cases, 90.1%), followed by bleeding from the gastrointestinal tract (4 cases, 5.6%), abdominal wall (1 case, 1.4%), intracranial vessels (1 case, 1.4%), and radial artery puncture site (1 case, 1.4%). Reintervention for hemostasis was required in 31 (43.7%) cases and blood transfused in 48 (67.6%) patients for active bleeding. Overall, 98 (10.4%) patients received postoperative blood transfusion in our cohort. Compared to non-bleeders, bleeding significantly extended length-of-stay (median (IQR), 12 (7–27) versus 7 (5–11) days, *p* < 0.001). Twelve (1.3%) perioperative mortalities were recorded, but none was related to VTE or bleeding.

Importantly, when compared with postoperative chemoprophylaxis, early administration significantly increased the risk of bleeding (OR 1.75, 95% CI 1.08–2.89, *p* = 0.038), particularly major bleeding following abdominal visceral resections (Table 2). Moreover, early chemoprophylaxis was associated with a significantly higher rate of blood transfusion, and reintervention for bleeding, as well as a greater fall in postoperative hemoglobin, compared with the postoperative chemoprophylaxis group (Table 2).

### Early Chemoprophylaxis Independently Predicts Postoperative Bleeding

Following univariate analysis, the factors that significantly correlated with postoperative bleeding included the following: higher ASA score, surgical case mix, a longer operative time, preoperative use of antiplatelet agents, lower baseline albumin level, and early chemoprophylaxis (Table 3). Of these, small bowel resection (OR 2.72, 95% CI 1.33–5.53, *p* = 0.006), preoperative use of antiplatelet agents (OR 1.73, 95% CI 1.04–2.87, *p* = 0.035), and early chemoprophylaxis (OR 1.93, 1.06–3.51, *p* = 0.032) were, on hierarchical multivariate analysis (treating hospital as a random effect), independent predictors of bleeding following abdominal visceral resections.

**Table 3:** Predictors of bleeding following major abdominal resections

Characteristics	Bleed *N* = 71	No Bleed *N* = 874	Univariate *p*-value	Multivariate		
				OR	95% CI	*p*-value
Demography						
Gender, male, *n* (%)	42 (59.2)	434 (49.7)	0.14	-	-	-
Age, mean (SD)	64.7 (14.2)	63.4 (12.8)	0.40	-	-	-
Body mass index (kg/m^2^), mean (SD)	28.0 (6.9)	28.3 (6.2)	0.69	-	-	-
Caprini score, median (IQR)	6 (6–8)	6 (5–7)	0.11	-	-	-
Operative						
Type, *n* (%)			**0.047**			
Esophagectomy	4 (5.6)	30 (3.4)		-	-	-
Gastrectomy	5 (7.0)	25 (2.9)		-	-	-
Splenectomy	0 (0.0)	13 (1.5)		-	-	-
Liver resection	9 (12.7)	124 (14.2)		-	-	-
Bile duct resection	2 (2.8)	10 (1.1)		-	-	-
Pancreatectomy	10 (14.1)	99 (11.3)		-	-	-
Small bowel resection	6 (8.5)	25 (2.9)		**2.72**	**1.33–5.54**	**0.006**
Colon resection	17 (23.9)	227 (26.0)		-	-	-
Rectal resection	18 (25.4)	292 (33.4)		-	-	-
Hartmann reversals	0 (0.0)	29 (3.3)		-	-	-
Surgery length (min), mean (SD)	350.5 (169.4)	280.7 (142.4)	** < 0.001**	-	-	-
Surgeon level, *n* (%)			0.57	-	-	-
Consultant	69 (97.2)	830 (95.1)				
Trainee	2 (2.8)	43 (4.9)				
Approach, *n* (%)			0.13	-	-	-
Open	33 (46.5)	322 (36.9)				
Laparoscopic	38 (53.5)	551 (63.1)				
Perioperative						
Malignant pathology, yes, *n* (%)	55 (77.5)	607 (69.5)	0.18	-	-	-
Therapeutic anticoagulant use, yes, *n* (%)	7 (9.9)	56 (6.4)	0.32	-	-	-
Antiplatelet agents use, yes, *n* (%)	16 (22.5)	116 (13.3)	**0.048**	**1.73**	**1.04–2.87**	**0.035**
ASA score, median (IQR)	3 (2–3)	3 (2–3)	**0.013**	-	-	-
Pre-op hemoglobin (g/L), mean (SD)	129.0 (17.5)	133.1 (18.5)	0.08	-	-	-
Pre-op platelet count, × 10^9^/L, mean (SD)	299.4 (141.2)	272.4 (95.9)	0.12	-	-	-
Pre-op INR, unit, mean (SD)	1.1 (0.1)	1.1 (0.1)	0.75	-	-	-
Pre-op bilirubin, mmol/L, mean (SD)	11.2 (12.3)	9.8 (12.7)	0.41	-	-	-
Pre-op albumin, g/L, mean (SD)	35.9 (5.1)	37.6 (5.7)	**0.023**	-	-	-
Mechanical prophylaxis use, yes, *n* (%)	70 (98.6)	871 (99.7)	0.27	-	-	-
Chemoprophylaxis type, LMWH, *n* (%)	59 (83.1)	781 (89.4)	0.12			
Chemoprophylaxis timing, early, *n* (%)	28 (39.4)	237 (27.1)	**0.038**	**1.93**	**1.06–3.51**	**0.032**

*ASA* American Society of Anesthesiology, *CI* confidence interval, *INR* international normalize ratio, *IQR* interquartile range, *LMWH* low molecular weight heparin, *OR* odds ratio, *SD* standard deviation

Bold indicates statistical significance

### Sensitivity Analysis for Bleeding and Clinical Venous Thromboembolism

Given that preoperative use of antiplatelet and anticoagulant agents as well as small bowel resection are independent predictors of bleeding, and may therefore confound the risk of postoperative bleeding and VTE, we performed a sensitivity analysis excluding these patients. This resulted in 213 (29.1%) and 520 (70.9%) patients receiving early and postoperative chemoprophylaxis, respectively. Consistent with our earlier analysis, clinical VTE rates were similar between these two groups (OR 1.97, 95% CI 0.60–6.58, *p* = 0.29). However, early chemoprophylaxis was associated with higher rates of bleeding (OR 1.82, 95% CI 1.01–3.28, *p* = 0.047), major bleeding (OR 1.96, 95% CI 1.01–3.81, *p* = 0.040), re-operative hemostasis (OR 2.28, 95% CI 0.96–5.16, *p* = 0.058), and blood transfusions (OR 1.76, 95% CI 1.07–2.89, *p* = 0.036) than those who received postoperative chemoprophylaxis (Table 4). Furthermore, another secondary hierarchical multivariate analysis included operation type as a random effect, to adjust for the possibility that timing of administration of chemoprophylaxis was dependent on this. Conclusions were again similar, with early chemoprophylaxis being associated with higher rates of bleeding (OR 1.82, 95% CI 1.10–3.01, *p* = 0.020), re-operative hemostasis (OR 2.97, 95% CI 1.44–6.12, *p* = 0.003), and blood transfusions (OR 2.02, 95% CI 1.29–3.16, *p* = 0.002). In this analysis, the odds of major bleeding were not significantly different between the groups.

**Table 4:** Bleeding and VTE outcomes excluding antiplatelet and anticoagulant agents and small bowel resection

Outcomes	Early *N* = 213	Post-op *N* = 520	Early vs. Post-op		
			OR	95% CI	*p*-value
All bleeding, *n* (%)	20 (9.1)	28 (5.2)	1.82	1.01–3.28	**0.047**
Major bleeding, *n* (%)	17 (7.7)	23 (4.3)	1.96	1.01–3.81	**0.040**
Minor bleeding, *n* (%)	3 (1.4)	5 (0.9)	1.47	0.39–5.57	0.70
Surgery for bleeding, *n* (%)	10 (4.5)	11 (2.0)	2.28	0.96–5.16	0.06
Blood transfusion for bleeders, *n* (%)	14 (6.4)	20 (3.7)	1.76	0.86–3.51	0.11
Blood transfusion overall, *n* (%)	28 (12.7)	41 (7.6)	1.76	1.07–2.89	**0.036**
Hb drop for bleeders, g/L, mean (SD)	− 32.0 (21.3)	− 29.7 (20.7)	-	-	0.71
Hb drop overall, g/L, mean (SD)	− 23.0 (15.4)	− 17.1 (15.3)	-	-	** < 0.001**
Venous thromboembolism, *n* (%)	4 (1.8)	5 (0.9)	1.97	0.60–6.56	0.29

*CI* confidence interval, *Hb* hemoglobin, *OR* odds ratio, *SD* standard deviation

Bold indicates statistical significance

## Discussion

This is the first multicenter study to comprehensively evaluate the timing of chemoprophylaxis and its effect on rates of bleeding and VTE after major abdominal visceral resection. Our data has shown increased rates of bleeding when chemoprophylaxis is administered early, prior to skin closure. Furthermore, it has also shown an association with higher rates of major bleeding and reintervention for bleeding. In comparison to postoperative administration, early chemoprophylaxis was also associated with a greater overall drop in hemoglobin, as well as higher rates of postoperative blood transfusion. Our study shows that in patients undergoing major abdominal visceral resection, early chemoprophylaxis is associated with increased complications, without additional appreciable benefit in VTE prophylaxis.

Incidence of clinical VTE (1.5%) and post operative bleeding (7.5%) in our cohort appears similar to that reported in international literature.^7,9,11^ Our data infers that chemoprophylaxis administered postoperatively is associated with a lower risk of bleeding, in comparison to early administration prior to skin closure. This appears congruent with findings from liver,^9^ gallbladder,^5^ breast,^14^ bariatric,^15^ ventral hernia,^16^ major joint,^17^ and spinal surgery.^18^ Three studies involving patients post pancreatectomy, duodenectomy, and liver surgery have reported that rates of VTE is higher when chemoprophylaxis was given postoperatively than when it was commenced prior to surgery.^8–10^ In contrast to our data, these studies reported on asymptomatic VTE, in smaller cohorts. This may explain their higher rates of VTE reported (10–20%) which is inconsistent with larger studies that describe symptomatic VTE.^19^ It is also difficult to interpret the clinical relevance of asymptomatic VTE in these populations. Moreover, randomized trials in colorectal and orthopedic surgery have reported that postoperative administration of chemoprophylaxis does not increase the risk of VTE.^7,17^ Our study contributes to a growing body of evidence to suggests that postoperative administration of chemoprophylaxis is a safer approach for patients, with similar risk of VTE, but lower rates of bleeding.

In addition to major bleeding and reintervention for bleeding, our data also showed that the early chemoprophylaxis group had a significantly greater fall in hemoglobin than those who received chemoprophylaxis postoperatively. This coincided with greater blood transfusion requirements in the early group, irrespectively of whether bleeding was clinically evident or not. This suggests that early chemoprophylaxis predisposes to developing “oozy wounds,” leading to occult blood loss. In light of increasing evidence suggesting that perioperative blood transfusion in cancer surgery is associated with poorer cancer-related outcomes, due to the immune suppressive effects of blood transfusion,^20,21^ minimizing occult blood loss is particularly important in our cohort, of which greater than 70% of patients underwent visceral resection for a malignancy.

The limitations of this study are related to its retrospective design. Firstly, we have addressed the imbalance between the groups using statistical sensitivity and subgroup analyses. Propensity score matching was also considered; however, given the relatively low proportion of bleeding and VTE events, this would not have yielded meaningful conclusions. Secondly, analysis of patient records requires a uniform approach to ensure data accuracy. This was maintained via several techniques. We used a universal data collection tool and provided standardized training sessions to investigators, to ensure correct interpretation of patient records. Parameters recorded were either binary or quantitative and included variables that are routinely recorded during patient admission. Patients that had < 95% of data collected were excluded from the study. This allowed for a data accuracy rate of 98%. Thirdly, our study only included clinical VTE, as asymptomatic cases were not routinely screened by radiological tests. Thus, the total incidence of VTE (clinical and asymptomatic) in our cohort is unknown. Furthermore, the incidence of both bleeding and VTE may have been underestimated. This is due to the potential for patients to present to other health services for care, external to where they had their initial operation. Despite this, reported rates of symptomatic VTE are reflected in similar studies, all bleeding cases were recorded in the same admission period, and all patients were followed-up. Finally, given the low rate of VTE seen in our cohort, our study is not powered to illustrate the true difference in symptomatic VTE.

## Conclusions

Patients undergoing major abdominal visceral resection are subject to variable timing of chemoprophylaxis administration. Rates of clinical VTE in this cohort are low. Early administration of chemoprophylaxis increases the risk of postoperative bleeding and its associated morbidity, without additional appreciable benefit in VTE prevention. Further studies will allow for evidence-based guidelines that may standardize the timing of perioperative VTE chemoprophylaxis, in patients undergoing major abdominal visceral resection.

## References

[1] Liu DS, Stevens S, Wong E, Fong J, Mori K, Fleming N (2020). Variations in practice of thromboprophylaxis across general surgical subspecialties: a multicentre (PROTECTinG) study of elective major surgeries. ANZ J Surg..

[2] Gould MK, Garcia DA, Wren SM, Karanicolas PJ, Arcelus JI, Heit JA (2012). Prevention of VTE in nonorthopedic surgical patients: Antithrombotic Therapy and Prevention of Thrombosis, 9th ed: American College of Chest Physicians Evidence-Based Clinical Practice Guidelines. Chest..

[3] Key NS, Khorana AA, Kuderer NM, Bohlke K, Lee AYY, Arcelus JI (2020). Venous Thromboembolism Prophylaxis and Treatment in Patients With Cancer: ASCO Clinical Practice Guideline Update. J Clin Oncol..

[4] Liu DS, Wong E, Fong J, Stevens S, Mori K, ProtectinG Investigators VC (2020). Perioperative thromboprophylaxis is highly variable in general surgery: results from a multicentre survey. ANZ J Surg..

[5] Liu DS, Stevens S, Wong E, Fong J, Mori K, Ward S (2020). Pre-operative and intra-operative chemical thromboprophylaxis increases bleeding risk following elective cholecystectomy: a multicentre (PROTECTinG) study. ANZ J Surg..

[6] Klifto KM, Gurno CF, Major M, Seal SM, Sacks JM, Rosson GD (2020). Pre-, intra-, and/or postoperative arterial and venous thromboembolism prophylaxis for breast surgery: Systematic review and meta-analysis. J Plast Reconstr Aesthet Surg..

[7] Zaghiyan KN, Sax HC, Miraflor E, Cossman D, Wagner W, Mirocha J (2016). Timing of Chemical Thromboprophylaxis and Deep Vein Thrombosis in Major Colorectal Surgery: A Randomized Clinical Trial. Ann Surg..

[8] Ainoa E, Uutela A, Nordin A, Makisalo H, Sallinen V (2021). Pre- vs. postoperative initiation of thromboprophylaxis in liver surgery. HPB (Oxford)..

[9] Doughtie CA, Priddy EE, Philips P, Martin RC, McMasters KM, Scoggins CR. Preoperative dosing of low-molecular-weight heparin in hepatopancreatobiliary surgery. Am J Surg. 2014;208(6):1009–15; discussion 15. doi:10.1016/j.amjsurg.2014.08.012. 10.1016/j.amjsurg.2014.08.012 25435300

[10] Reinke CE, Drebin JA, Kreider S, Kean C, Resnick A, Raper S (2012). Timing of preoperative pharmacoprophylaxis for pancreatic surgery patients: a venous thromboembolism reduction initiative. Ann Surg Oncol..

[11] Kakkar AK, Agnelli G, Fisher W, George D, Lassen MR, Mismetti P (2014). Preoperative enoxaparin versus postoperative semuloparin thromboprophylaxis in major abdominal surgery: a randomized controlled trial. Ann Surg..

[12] Clavien PA, Barkun J, de Oliveira ML, Vauthey JN, Dindo D, Schulick RD (2009). The Clavien-Dindo classification of surgical complications: five-year experience. Ann Surg..

[13] Bahl V, Hu HM, Henke PK, Wakefield TW, Campbell DA, Caprini JA (2010). A validation study of a retrospective venous thromboembolism risk scoring method. Ann Surg..

[14] Keith JN, Chong TW, Davar D, Moore AG, Morris A, Gimbel ML (2013). The timing of preoperative prophylactic low-molecular-weight heparin administration in breast reconstruction. Plast Reconstr Surg..

[15] Altieri MS, Yang J, Hajagos J, Spaniolas K, Park J, Gasparis AP (2018). Evaluation of VTE prophylaxis and the impact of alternate regimens on post-operative bleeding and thrombotic complications following bariatric procedures. Surg Endosc..

[16] PROTECTinG investigators, VERITAS Collaborative. Chemical thromboprophylaxis before skin closure increases bleeding risk following major ventral hernia repair: A multicenter cohort study. Surgery. 2022. 10.1016/j.surg.2022.01.023. 10.1016/j.surg.2022.01.023 35248362

[17] Hull RD, Pineo GF, Francis C, Bergqvist D, Fellenius C, Soderberg K (2000). Low-molecular-weight heparin prophylaxis using dalteparin in close proximity to surgery vs warfarin in hip arthroplasty patients: a double-blind, randomized comparison. The North American Fragmin Trial Investigators. Arch Intern Med..

[18] Khan M, Jehan F, O'Keeffe T, Hamidi M, Truitt M, Zeeshan M (2018). Optimal Timing of Initiation of Thromboprophylaxis after Nonoperative Blunt Spinal Trauma: A Propensity-Matched Analysis. J Am Coll Surg..

[19] Baltatzis M, Low R, Stathakis P, Sheen AJ, Siriwardena AK, Jamdar S (2017). Efficacy and safety of pharmacological venous thromboembolism prophylaxis following liver resection: a systematic review and meta-analysis. HPB (Oxford)..

[20] Goubran HA, Elemary M, Radosevich M, Seghatchian J, El-Ekiaby M, Burnouf T (2016). Impact of Transfusion on Cancer Growth and Outcome. Cancer Growth Metastasis..

[21] Wu HL, Tai YH, Lin SP, Chan MY, Chen HH, Chang KY (2018). The Impact of Blood Transfusion on Recurrence and Mortality Following Colorectal Cancer Resection: A Propensity Score Analysis of 4,030 Patients. Sci Rep..

